# Supplementary material for: Identification of photoperiod-induced specific miRNAs in the adrenal glands of Sunite sheep (Ovis aries)
Source: Front Vet Sci. 2022 Jul 22;9:888207. doi: 10.3389/fvets.2022.888207 (PMC9354845; doi:10.3389/fvets.2022.888207)
Supplement: Supplementary file 1 [file Data_Sheet_1.ZIP › Supplementary file/miRDeep2 file.zip!/miRDeep2 file/Know_Structure/LP42A3/oar-mir-370.pdf]

miRBase precursor : oar-mir-370
Total read count : 18804
oar-miR-370-5p read count : 65
oar-miR-370-3p read count : 18720
remaining reads : 19

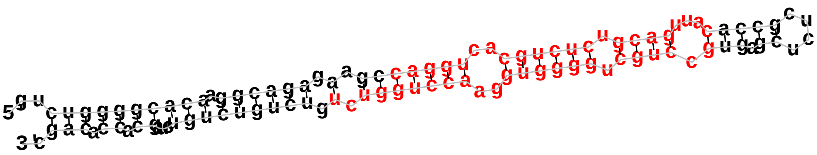

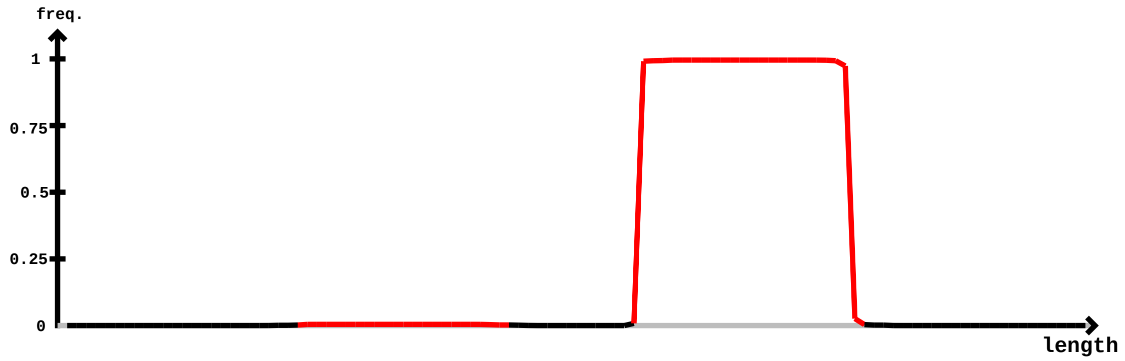


```
                                                                        oar-miR-370-3p
                                 oar-miR-370-5p
5'- gucuggggcacaaggcagagaagccaggucacgucucugcaguuacaccgcucucgagugccugcuggggguggaaccuggucugucugucugucuagcaccacagc   -3'    exp
    ..((((((((((.((((((.(.(((((((..((((((.((((...(((((....)).))).))))..))))))..))))))).).)))))))))...)).)).))).    reads   mm   sample
    ....................aagccaggucacgucucugcagu................................................................    1       0    seq
    .....................agccaggucacgucucugcag.................................................................    1       0    seq
    .....................agccaggucacgucucugcagu................................................................    15      0    seq
    .....................agccaggucacgucucugcaguu...............................................................    2       0    seq
    .......................ccaggucacgucucugcagu................................................................    2       0    seq
    .......................ccaggucacgucucugcaguu...............................................................    15      0    seq
    .......................ccaggucacgucucugcaguuac.............................................................    1       0    seq
    ........................caggucacgucucugcaguu...............................................................    4       0    seq
    ........................caggucacgucucugcaguua..............................................................    2       0    seq
    ........................caggucacgucucugcaguuac.............................................................    15      0    seq
    ........................caggucacgucucugcaguuaca............................................................    18      0    seq
    ........................caggucacgucucugcaguuacac...........................................................    7       0    seq
    .........................aggucacgucucugcaguuacac...........................................................    1       0    seq
    ..........................................................ugccugcuggggguggaaccu............................    1       0    seq
    ..........................................................ugccugcuggggguggaaccug...........................    1       0    seq
    ..........................................................ugccugcuggggguggaaccugg..........................    7       0    seq
    ..........................................................ugccugcuggggguggaaccuggu.........................    143     0    seq
    ..........................................................ugccugcuggggguggaaccugguc........................    3       0    seq
    ..........................................................ugccugcuggggguggaaccuggucu.......................    1       0    seq
    ...........................................................gccugcuggggguggaacU.............................    1       1    seq
    ...........................................................gccugcuggggguggaacc.............................    2       0    seq
    ...........................................................gccugcuggggguggaaccu............................    7       0    seq
    ...........................................................gccugcuggggguggaaccug...........................    33      0    seq
    ...........................................................gccugcuggggguggaaccugg..........................    360     0    seq
    ...........................................................gccugcuggggguggaaccuggu.........................    17651   0    seq
    ...........................................................gccugcuggggguggaaccugguc........................    404     0    seq
    ...........................................................gccugcuggggguggaaccuggucu.......................    26      0    seq
    ............................................................ccugcuggggguggaaccug...........................    1       0    seq
    ............................................................ccugcuggggguggaaccugg..........................    1       0    seq
    ............................................................ccugcuggggguggaaccuggu.........................    15      0    seq
    ............................................................ccugcuggggguggaaccugguc........................    12      0    seq
    ............................................................ccugcuggggguggaaccuggucu.......................    3       0    seq
    .............................................................cugcuggggguggaaccuggu.........................    7       0    seq
    .............................................................cugcuggggguggaaccugguc........................    4       0    seq
```

```
                                                                        oar-miR-370-3p
                        oar-miR-370-5p
gucuggggcacaaggcagagaagccaggucacgucucugcaguuacaccgcucucgagugccugcugggguggaaccuggucugucugucugucuagcaccacagc

.............................................................cugcugggguggaaccuggucu.......................   3     0     seq
..............................................................ugcugggguggaaccuggucug......................   2     0     seq
..............................................................ugcugggguggaaccuggucugu.....................  28     0     seq
..............................................................ugcugggguggaaccuggucuguc....................   2     0     seq
...............................................................gcugggguggaaccuggucugu.....................   2     0     seq
```
